# Supplementary material for: The interactome of intact mitochondria by cross-linking mass spectrometry provides evidence for coexisting respiratory supercomplexes
Source: Mol Cell Proteomics. 2017 Dec 8;17(2):216–32. doi: 10.1074/mcp.RA117.000470 (PMC5795388; doi:10.1074/mcp.RA117.000470)
Supplement: Supplemental Data [file supp_RA117.000470_133922_0_supp_23978_fzffwf.zip › spectra_annotation/mito_DR_spectra_annotation/377-1-3-1-3-1.pdf]

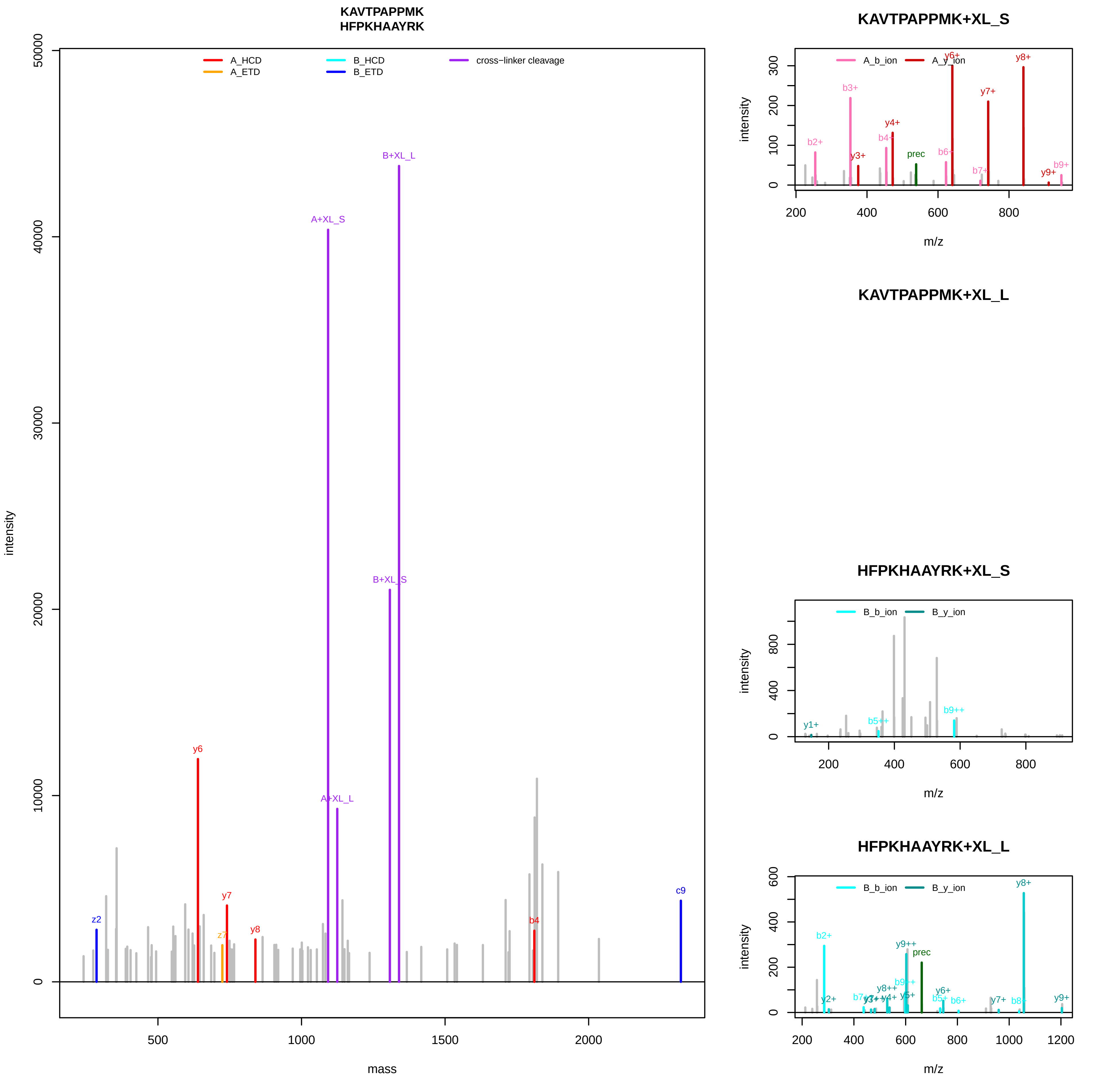

KAVTPAPPMK
HFPKHAAYRK
A_HCD
A_ETD
B_HCD
B_ETD
cross−linker cleavage
intensity
mass
z2
y6
z7
y7
y8
A+XL_S
A+XL_L
B+XL_S
B+XL_L
b4
c9
KAVTPAPPMK+XL_S
A_b_ion
A_y_ion
b2+
b3+
y3+
b4+
y4+
prec
b6+
y6+
b7+
y7+
y8+
y9+
b9+
m/z
KAVTPAPPMK+XL_L
HFPKHAAYRK+XL_S
B_b_ion
B_y_ion
y1+
b5++
b9++
HFPKHAAYRK+XL_L
b2+
y2+
b7+
y3+
y8++
y4+
y9++
b9++
y5+
prec
b5+
y6+
b6+
y7+
b8+
y8+
y9+
